# Association of Visual Display Terminal Usage with Self-Rated Health and Psychological Distress among Japanese Office Workers during the COVID-19 Pandemic

**DOI:** 10.3390/ijerph18179406

**Published:** 2021-09-06

**Authors:** Yu Par Khin, Yusuke Matsuyama, Takahiro Tabuchi, Takeo Fujiwara

**Affiliations:** 1 Department of Global Health Promotion, Tokyo Medical and Dental University, Tokyo 113-8510, Japan; yukhin.hlth@tmd.ac.jp (Y.P.K.); matsuyama-thk@umin.org (Y.M.); 2 Cancer Control Center, Osaka International Cancer Institute, Osaka 541-8567, Japan; tabuchitak@gmail.com

**Keywords:** screen time, mental health, physical health, occupational health, workplace

## Abstract

The aim of this study was to examine the association of the duration of visual display terminal (VDT) usage for work and non-work activities with self-rated health (SRH) and psychological distress among office workers during the COVID-19 pandemic in Japan. A cross-sectional data of 7088 office workers from a web-based, self-administered survey conducted from 25 August 2020, to 30 September 2020, was used. Multiple logistic regression analysis was applied. Compared to those who used a VDT for 4–9 h for work, office workers who used a VDT for ≥10 h for work had poor SRH (odds ratio (OR): 1.65; 95% confidence interval (CI): 1.13, 2.41) and severe psychological distress (OR: 2.23; 95% CI: 1.52, 3.28). VDT usage for less than 1 h (OR: 1.37, 95% CI: 1.12, 1.67) and 1–3 h (OR: 1.42, 95% CI: 1.12, 1.80) for work were also associated with severe psychological distress. Stratification analysis by age showed a significant association of VDT usage for work with poor SRH among 30–64-year-olds, while a U-shape association was found between VDT usage for work and psychological distress with the younger age group (15–29 years old). During the COVID-19 pandemic in Japan, the prolonged usage of VDT for work can deteriorate both general and psychological health, while moderate usage of VDT for work can reduce psychological distress.

## 1. Introduction

The new coronavirus disease 2019 (COVID-19) was first detected in December 2019 and has since spread across the world. As there are no specific treatment and prevention programs to address the newly evolved disease, the World Health Organization (WHO) has recommended non-pharmacological measures to control the spread of the infection [1], and such measures were launched in Japan on 25 February 2020. These policies, including social distancing and encouraging telework, have brought significant changes to society [2]. The telework culture has led to an increased use of visual display terminals (VDTs), such as computers, smartphones, and tablets, for work among office workers [3]. Moreover, studies in Japan and elsewhere have shown that, during the lockdown or relevant measures such as limiting social outings and events, daily average screen time has increased [4,5] as well as VDT usage and screen time for work [3,6].

VDT usage and length of screen time have been associated with poor self-rated health and psychological distress even before the COVID-19 pandemic. A Japanese study proved that VDT usage of more than 5 h was associated with poor mental health among administrative workers [7]. Additionally, a national study of adults in Saudi Arabia showed that more than 3 h of screen time daily led to poor self-rated health [8]. Similar findings have been reported during the COVID-19 pandemic. A Canadian study reported that limited screen time improved general and mental health during lockdown [4], while increased screen time had been associated with an increased risk of depression, stress, and loneliness among adults in the US [5]. However, few studies have been conducted among office workers, who are most likely to be affected by the policies encouraging social distancing and teleworking during the COVID-19 pandemic [3,6]. These studies for office workers only focused on increased VDT usage during the lockdown, which was not implemented in Japan, and the evidence is still lacking as to whether VDT usage has an association with physical and mental health during the COVID-19 pandemic. Though the association with VDT usage and health varies with the purpose of use and age groups [9,10], and the increased screen time varies with age during the COVID-19 pandemic [11,12], the studies of VDT usage by office workers have not differentiated between work and non-work or stratified by age [3,4,5,6]. Therefore, the aim of this study was to determine the association between work and non-work VDT usage time and self-rated health and psychological distress among office workers in Japan during the COVID-19 pandemic. Stratification analysis by age was also performed to evaluate whether the impact of VDT usage varied by age group.

## 2. Materials and Methods

### 2.1. Study Participants

Cross-sectional data was used from the Japan “COVID-19 and Society” Internet Survey (JACSIS) study, which was a web-based, self-administered survey across Japan. The invitation email was distributed to 224,389 panelists of Rakuten insight, a large internet research company in Japan, between 25 August 2020 and 30 September 2020. They were selected by simple random sampling to represent the Japanese population according to gender, age and prefecture of residence. Thus, panelists of this study covered workers from several occupations in companies or who were self-employed. The survey continued until the number of respondents reached the targeted sample size for each gender, age, and prefecture (*n* = 28,000), which was predetermined according to the population distribution in 2019. Out of 28,000, those who gave inappropriate answers (i.e., those whom we speculated not reading the questionnaire; *n* = 2518) were excluded by checking whether answers were consistent with a developed algorithm [13,14]. In the algorithm, those who failed to respond to our dummy question, “Please choose the second item from the bottom of a list”, and who chose every item in the questions “Select which drugs are used”, with a list of 7 substances, and “Choose which chronic diseases apply”, with a list of 16 diseases, were excluded. Further, to focus on office workers, students (*n* = 1751), retirees (*n* = 1065), housewives (*n* = 4197), unemployed people (*n* = 3015), those who had jobs that required interacting with people (*n* = 3793), manual laborers (*n* = 4163), and those who were absent for the main exposure variable (*n* = 410) were excluded, so that the samples were eligible for the present study’s purpose, which was to determine the association of VDT usage and health among Japanese office workers. Finally, 7088 office workers were included in the analysis. The study design was approved by the Institutional Review Board of the Osaka International Cancer Institute (No. 20084).

### 2.2. Measurements

For the outcome variable, self-rated health status was measured by asking the respondents, “How is your current health status?”. For analytical purposes, we created a dichotomous variable in which the self-rated health status classified either “poor” or “fair” as poor health, and “good”, “very good”, or “excellent” as non-poor health. These categories of self-rated health have been used in previous research and can independently predict mortality [15,16]. For psychological distress, we used the validated Japanese version of the K6 psychological screening tool for anxiety and mood disorder [17]. Respondents with a total score of 13 or more were classified as having severe psychological distress [17].

To assess VDT usage, the respondents were asked, “From June to now, how much time per day (on average) did you spend on VDT usage for work and non-work activities, respectively?” with options of 0 h, less than 30 min, 30 min, 1, 2, 3, 4–5, 6–7, 8–9, 10–11, 12 h and above per day, or do not know. The VDT usage from June to September 2020 was categorized as less than 1, 1–3, 4–9, and 10 h or more per day to pronounce the severity of the impact at different times. VDT usage for non-working activities was categorized as less than 1, 1–3, 4–5, and 6 h or more per day as the duration of VDT usage for non-working activities was shorter than that for work among the sample. VDT usage had mainly been assessed via subjective measurements [18], and a similar questionnaire was used to assess VDT usage [19].

Other covariates included demographic factors (age and gender), socioeconomic background (education and income), working hours in the previous month, body mass index (BMI), hospitalization history from April 2020, presence or absence of depression and anxiety, and past medical history (self-reported hypertension, diabetes, chronic asthma, cancer, heart disease (angina and myocardial infarction), chronic obstructive pulmonary disease (COPD), and stroke).

### 2.3. Statistical Analysis

Chi-square test and multiple logistic regression were used for the analysis. To evaluate the health status of VDT users by comparing with the most significant population, 4–9 h of VDT usage per day for work and less than 1 h of VDT usage per day not for work were used as the reference category. For self-rated health, Model 1 was adjusted for age, gender, education, income, BMI, history of hospitalization, anxiety and depression, and past medical history. Model 2, in addition to Model 1, was adjusted for the working hours in the previous month. For psychological distress, the same covariates, except for the presence of anxiety and depression, were adjusted. Stratified analyses by age for the association between VDT usage for work and both self-rated health status and psychological distress were also conducted. The age groups were classified as 15–29 years, 30–64 years, and 65 years and older. *p* for trend was calculated for each model. Stata software version 15.0 from StataCorp LLC (College Station, TX, USA) was used for the analysis.

## 3. Results

Table 1 shows the characteristics of the participants by self-rated health and psychological distress. Of the 7088 respondents, 12.9% reported having poor self-rated health. Participants of the analytical sample were mainly 30–59 years old (77.3%), male (60.2%), university graduates (62.4%), had an income of Japanese Yen (JPY) 5 to 10 million per year (40.1%), BMI of 18.5 to 22.9 (51.2%), no history of hospitalization from April 2020 (96.9%), no history of anxiety and depression (72.7%), and had hypertension as the most common chronic disease (20.8%). They tended to spend 30–49 working hours per week (65.6%), 4–9 h using a VDT for work per day (47.6%), and less than 1 h using a VDT not for work per day (51.9%) (Table 1).

Respondents with poor self-rated health were likely to be middle-aged (30–64 years old), had low education and income, had a BMI of 27 kg/m^2^ or higher, had a history of hospitalization, anxiety, depression, or chronic illness, worked more than 50 h per week, and used VDT for work less than 1 h per day or more than 10 h per day. In terms of psychological distress, 9.1% of the respondents reported having severe psychological distress and were more likely to be younger in age, female, low-income, have a BMI < 18.5 kg/m^2^, a history of hospitalization or chronic illnesses other than hypertension, worked 0 to 29 h or more than 50 h per week, and worked with VDTs for less than 1 h per day or more than or equal 10 h per day.

Table 2 summarizes the outcome of logistic regression for poor self-rated health and VDT usage from June to September. Participants who used VDT ≥ 10 h for work per day were 1.65 times more likely to show poor self-rated health (OR = 1.65, 95% CI 1.13–2.41) after adjusting for age, gender, education, income, BMI, hospitalization, anxiety and depression, past medical history, and working hours in the previous month. On the contrary, participants who used a VDT for less than 1 h (OR = 1.08, 95% CI 0.91, 1.30) and 1–3 h (OR = 0.99, 95% CI 0.8, 1.23) for work per day were not significantly associated with poor self-rated health. VDT for non-working activities was not associated with self-rated health. *p* for trend for both VDT for work (*p* for trend = 0.09) and not for work (*p* for trend = 0.15) were not significant.

Table 3 summarizes the outcome of logistic regression for severe psychological distress and VDT usage from June to September. Participants who used VDT for less than 1 h for work per day were 1.37 times (OR = 1.37, 95% CI 1.12, 1.67), and those who used VDT 1–3 h for work per day were 1.42 times (OR = 1.42, 95% CI 1.12, 1.80) more likely to have severe psychological distress (after adjusting for age, gender, education, income, BMI, hospitalization, past medical history, and working hours in the previous month). Those who used a VDT for ≥10 h for work per day were 2.23 times more likely to have severe psychological distress (OR = 2.23, 95% CI 1.52, 3.28) and *p* for trend was not significant (*p* for trend = 0.11). Although any VDT categories for non-work activities did not show significant associations with psychological distress, the results from *p* for trend suggested linear positive association after adjustment of working hours (*p* for trend = 0.04).

The associations between VDT usage for work and poor self-rated health status (Table 4) and severe psychological distress (Table 5) by age groups are shown. Using a VDT for ≥10 h for work per day was 1.88 times more likely to be associated with poor self-rated health among people aged 30–64 years old (OR 1.88, 95% CI 1.25, 2.85), but not for the younger age group. *p* for trend was not significant for all age groups. For severe psychological distress, using VDT < 1 h for work per day was 1.92 times (OR 1.92, 95% CI 1.20, 3.06), and using VDT 1–3 h for work per day was 2.54 times (OR 2.54, 95% CI 1.48, 4.35) more likely to be associated with severe psychological distress among participants 15–29 years old. Using VDT ≥ 10 h for work per day was not significantly associated with severe psychological distress for 15–29 years old (OR 2.27, 95% CI 0.94, 5.47). Participants 30–64 years old were 2.33 times more likely to be associated with severe psychological distress when using a VDT for ≥10 h for work per day (OR 2.33, 95% CI 1.52, 3.59). *p* for trend was significant for severe psychological distress and VDT for work among participants of 15–29 years old. (*p* for trend = 0.049).

## 4. Discussion

This study was conducted to find the cross-sectional association between VDT usage and self-rated health and psychological distress among Japanese office workers during the COVID-19 pandemic. The usage of VDT for work for 10 h and above per day was found to be associated with poor self-rated health and severe psychological distress among the middle age group. Additionally, apart from the previous findings, which proved the negative health association with VDT or screen time, 4–9 h usage of VDT for work during the COVID-19 pandemic was the least likely to be associated with severe psychological distress among younger age office workers. VDT for non-work activities was not significantly associated with both physical and mental health. Although the association of VDT usage or screen time with physical and mental health has long been demonstrated [10,20], this is the first study that differentiates between work and non-work-related usage of VDT among office workers during the COVID-19 pandemic.

Previous studies showed a positive association between the usage of VDTs and physical and mental health in adults [18]. This association is mainly explained by the fact that sedentary behavior at work leads to obesity, which affects physical and psychological health [21], and blue light exposure disrupts circadian rhythms [22]. Although the disruption of rhythms caused by blue light exposure does not change with age [23], in the adult population, middle-aged and older people seem to be less physically active [21,24], and this was also the case during the COVID-19 pandemic [25]. In addition, using VDTs for more than 10 h a day may be associated with demanding work and low job control, which deteriorates general and psychological health [26]. Therefore, reduced physical activity and the demanding work of individuals with longer VDT usage during the COVID-19 pandemic would explain our results.

Apart from the previous studies, the current study found that moderate usage of VDT was associated with lower psychological distress, especially among the younger age group (15–29 years old). Social distancing and teleworking are proven to prevent COVID-19 infection, providing psychological relief to Japanese office workers [27]. This association is more pronounced in younger generations, as they are more accustomed to using technological devices [28]. Moreover, the economic impact and reduction in the workforce due to COVID-19 has psychological consequences [29], and young people are disproportionately affected by the reduction in working hours during the pandemic [30]. Moderate usage of VDTs may be associated with the privilege of being able to work from home and be safe from a diminished workforce.

According to our study, VDT usage for non-working activities was not significantly associated with SRH but had a linear association with psychological distress. On the other hand, moderate VDT usage for work was associated with less psychological distress. The difference in the association between work and non-work usage with psychological distress may be due to the fact that being employed is psychologically beneficial by increasing self-reliance [31], whereas non-work VDT usage has little psychological value. During the COVID-19 pandemic, physical distancing makes VDTs a means of social interaction [32], which is proven to be associated with improved physical and psychological health [33]. However, in our study, VDT usage for non-work activities was not associated with psychological distress, and there was a potential negative linear association. This may suggest that the study participants used VDT for non-work activities without interacting with others. Further research detailing screen use among office workers is needed to assess the health effects of different uses of screens.

### Limitations

There are some limitations to this study. First, the measurement of VDT usage is subjective and may not reflect actual usage. However, VDT usage was estimated by asking the total hours of VDT usage per day, which is a closed estimate of actual usage [34]. Second, participants may not be able to differentiate the purpose of VDT usage for work and not for work. Criteria or a set of rules to differentiate between the purposes of usage, such as asking working hours first, then asking the percentage of VDT usage during working hours, may be needed. Therefore, further studies should be conducted with more detailed measurements of VDT usage in and out of work. Thirdly, because this study is cross-sectional, the causal relationship remains unknown, and we do not know if the effects of VDT usage during the COVID-19 pandemic will persist in the long term. Further studies are needed to detect the long-term health effects of non-pharmacological measures during the COVID-19 pandemic. Finally, Japan is a country with relatively long working hours [35] when compared to other developed countries, and our result may not be applicable in those countries where VDT usage for more than 10 h per day for work is exceptional.

## 5. Conclusions

This study shows that prolonged VDT usage for work has a significant impact on the health of office workers over 30 years old, and thus, prolonged VDT usage for work should be avoided. Moderate VDT usage is associated with reduced psychological distress in young people. Therefore, moderate usage should be promoted, especially among young office workers. Under social distancing measures, VDT usage for work and outside work were found to have different associations with physical and psychological health. Further research on the relationship between purpose-specific VDT usage and health is recommended.

## Figures and Tables

**Table 1:** Self-rated health and psychological distress by demographic characteristics of participants (*n* = 7088).

Variables	Total Participants	Self-Rated Health		*p*-Value	Severe Psychological Distress		*p*-Value
		Non-Poor	Poor		No (K6 < 13)	Yes (K6 ≥ 13)	
	(*n* = 7088)	(*n* = 6171, 87.1%)	(*n* = 917, 12.9%)		(*n* = 6445; 90.9%)	(*n* = 643; 9.1%)	
	*n* (%)	*n* (%)	*n* (%)		*n* (%)	*n* (%)	
Age (years)							
15–29	955 (13.5%)	859 (13.9%)	96 (10.5%)	<0.001	800 (12.4%)	155 (24.1%)	<0.001
30–64	5480 (77.3%)	4723 (76.5%)	757 (82.6%)		5007 (77.7%)	473 (73.6%)	
≥65	653 (9.2%)	589 (9.5%)	64(7.00%)		638 (9.9%)	15(2.3%)	
Gender							
Male	4265 (60.2%)	3701 (60%)	564 (61.5%)	0.380	3904 (60.6%)	361 (56.1%)	0.030
Female	2823 (39.8%)	2470 (40%)	353 (38.5%)		2541 (39.4%)	282 (43.9%)	
Education							
High School and below	1309 (18.5%)	1115 (18.1%)	194 (21.2%)	0.009	1198 (18.6%)	111 (17.3%)	0.66
College, vocational school, and others	1355 (19.1%)	1163 (18.9%)	192 (20.9%)		1234 (19.2%)	121 (18.8%)	
University and above	4424 (62.4%)	3893 (63.1%)	531 (57.9%)		4013 (62.3%)	411 (63.9%)	
Income (Million Yen, yearly as of 2019)							
0–5	2123 (30%)	1772 (28.7%)	351 (38.3%)	<0.001	1854 (28.8%)	269 (41.8%)	<0.001
5–9	2843 (40.1%)	2514 (40.7%)	329 (35.9%)		2616 (40.6%)	227 (35.3%)	
≥10	1141 (16.1%)	1025 (16.6%)	116 (12.7%)		1061 (16.5%)	80 (12.4%)	
Not answered or not know	981 (13.8%)	860 (13.9%)	121 (13.2%)		914 (14.2%)	67 (10.4%)	
BMI (kg/m^2^)							
<18.5	763 (10.8%)	657 (10.7%)	106 (11.6%)	<0.001	657 (10.2%)	106 (16.5%)	<0.001
18.5–22.9	3626 (51.2%)	3224 (52.2%)	402 (43.8%)		3289 (51%)	337 (52.4%)	
23–26.9	1967 (27.8%)	1707 (27.7%)	260 (28.4%)		1832 (28.4%)	135 (21%)	
≥27	732 (10.3%)	583 (9.5%)	149 (16.3%)		667 (10.4%)	65 (10.1%)	
History of hospitalization							
Present	220 (3.1%)	175 (2.8%)	45 (4.9%)	0.001	171 (2.7%)	49 (7.6%)	<0.001
Absent	6868 (96.9%)	5996 (97.2%)	872 (95.1%)		6274 (97.4%)	594 (92.4%)	
Anxiety and depression							
Present	1939 (27.4%)	1356 (22%)	583 (63.6%)	<0.001			
Absent	5149 (72.7%)	4815 (78%)	334 (36.4%)				
Past medical history							
Hypertension (Yes)	1471 (20.8%)	1177 (19.1%)	294 (32.1%)	<0.001	1321 (20.5%)	150 (23.3%)	0.09
Diabetes (Yes)	504 (7.1%)	381 (6.2%)	123 (13.4%)	<0.001	426 (6.61%)	78 (12.1%)	<0.001
Asthma (Yes)	962 (13.6%)	780 (12.6%)	182 (19.9%)	<0.001	805 (12.5%)	157 (24.4%)	<0.001
Cancer (Yes)	332 (4.7%)	257 (4.2%)	75 (8.2%)	<0.001	278 (4.3%)	54 (8.4%)	<0.001
Heart disease (Yes)	234 (3.3%)	181 (2.9%)	53 (5.8%)	<0.001	181 (2.8%)	53 (8.24%)	<0.001
COPD (Yes)	114 (1.6%)	86 (1.4%)	28 (3.1%)	<0.001	73 (1.1%)	41 (6.4%)	<0.001
Stroke (Yes)	147 (2.1%)	119 (1.9%)	28 (3.1%)	0.03	105 (1.6%)	42 (6.5%)	<0.001
Working hours in the previous month (hours in a week)							
0–29	1530 (21.6%)	1323 (21.4%)	207 (22.6%)	0.03	1363 (21.2%)	167 (26%)	<0.001
30–49	4646 (65.6%)	4076 (66.1%)	570 (62.2%)		4277 (66.4%)	369 (57.4%)	
≥50	912 (12.9%)	772 (12.5%)	140 (15.3%)		805 (12.5%)	107 (16.6%)	
Visual Display Terminal (VDT) usage for work (per day)							
<1 h	2202 (31.1%)	1909 (30.9%)	293 (32%)	0.004	1971 (30.6%)	231 (35.9%)	<0.001
1–3 h	1266 (17.9%)	1111 (18%)	155 (16.9%)		1139 (17.7%)	127 (19.8%)	
4–9 h	3372 (47.6%)	2953 (47.9%)	419 (45.7%)		3130 (48.6%)	242 (37.6%)	
≥10 h	248 (3.5%)	198 (3.2%)	50 (5.5%)		205 (3.2%)	43 (6.7%)	
VDT usage not for work (per day)							
<1 h	3679 (51.9%)	3232 (52.4%)	447 (48.8%)	0.06	3362 (52.2%)	317 (49.3%)	0.09
1–3 h	2825 (39.9%)	2445 (39.6%)	380 (41.4%)		2568 (39.8%)	257 (40%)	
4–5 h	319 (4.5%)	275 (4.5%)	44 (4.8%)		283 (4.4%)	36 (5.6%)	
≥6 h	265 (3.7%)	219 (3.6%)	46 (5%)		232 (3.6%)	33 (5.1%)	

Abbreviations: body-mass index, BMI, chronic obstructive pulmonary disease, COPD, visual display terminal, VDT.

**Table 2:** Association between the VDT usage and poor self-rated health.

VDT Usage from June to September (per Day)	Model 1	Model 2
	OR (95% CI)	OR (95% CI)
For work		
<1 h	1.09 (0.91, 1.29)	1.08 (0.91, 1.30)
1–3 h	0.99 (0.8, 1.23)	0.99 (0.8, 1.23)
4–9 h	ref	ref
≥10 h	1.75 (1.22, 2.51)	1.65 (1.13, 2.41)
*p* for trend	0.80	0.09
Not for work		
<1 h	ref	ref
1–3 h	1.05 (0.89, 1.23)	1.05 (0.89, 1.23)
4–5 h	1.16 (0.81, 1.66)	1.15 (0.80, 1.65)
≥6 h	1.28 (0.89, 1.84)	1.28 (0.89, 1.83)
*p* for trend	0.15	0.15

Abbreviations: odds ratio, OR, confidence interval, CI, reference, ref. Model 1 adjusted for age, gender, education, income, BMI, hospitalization, anxiety and depression, and past medical history. Model 2: model 1 + working hours in the previous month. VDT for work and not for work were separately included to the models.

**Table 3:** Association between the VDT usage and severe psychological distress.

VDT Usage from June to September (per Day)	Model 1	Model 2
	OR (95% CI)	OR (95% CI)
For work		
<1 h	1.43 (1.18, 1.74)	1.37 (1.12, 1.67)
1–3 h	1.47 (1.16, 1.86)	1.42 (1.12, 1.80)
4–9 h	ref	ref
≥10 h	2.66 (1.85, 3.83)	2.23 (1.52, 3.28)
*p* for trend	0.08	0.11
Not for work		
<1 h	ref	ref
1–3 h	1.07 (0.89, 1.27)	1.07 (0.90, 1.28)
4–5 h	1.39 (0.95, 2.02)	1.37 (0.94, 2.00)
≥6 h	1.44 (0.97, 2.13)	1.41 (0.95, 2.09)
*p* for trend	0.03	0.04

Abbreviations: odds ratio, OR, confidence interval, CI, reference, ref. Model 1 adjusted for age, gender, education, income, BMI, hospitalization, and past medical history. Model 2: model 1 + working hours in the previous month.

**Table 4:** Association between the VDT usage for work and self-rated health stratified by age groups.

VDT Usage for Work	Age (Years)		
	15–29	30–64	≥65
<1 h	1.52 (0.89, 2.59)	1.02 (0.83, 1.25)	1.03 (0.49, 2.14)
1–3 h	0.82 (0.38, 1.79)	1.02 (0.80, 1.30)	0.99 (0.46, 2.14)
4–9 h	ref	ref	ref
≥10 h	0.70 (0.18, 2.67)	1.88 (1.25, 2.85)	NA
*p* for trend	0.08	0.35	0.93

Abbreviations: reference, ref. Adjusted for gender, education, income, BMI, hospitalization, and past medical history and working hours in the previous month.

**Table 5:** Association between the VDT usage for work and severe psychological distress stratified by age groups.

VDT Usage for Work	Age (Years)		
	15–29	30–64	≥65
<1 h	1.92 (1.20, 3.06)	1.26 (0.99, 1.59)	0.89 (0.23, 3.42)
1–3 h	2.54 (1.48, 4.35)	1.29 (0.99, 1.70)	0.49 (0.08, 3.05)
4–9 h	ref	ref	ref
≥10 h	2.27 (0.94, 5.47)	2.33 (1.52, 3.59)	NA
*p* for trend	0.049	0.55	0.94

Abbreviations: reference, ref. Adjusted for gender, education, income, BMI, hospitalization, past medical history, and working hours in the previous month.

## Data Availability

The datasets used and analyzed during the current study are available from the corresponding author on reasonable request.
